# Universal transient radiation dynamics by abrupt and soft temporal transitions in optical waveguides

**DOI:** 10.1515/nanoph-2024-0525

**Published:** 2025-01-06

**Authors:** Amir Shlivinski, Yakir Hadad

**Affiliations:** School of Electrical and Computer Engineering, Ben-Gurion University, Beer Sheva, Israel; School of Electrical Engineering, Tel-Aviv University, 69978, Tel-Aviv, Israel

**Keywords:** temporal transitions, transient radiation, time-varying media, chirped radiation

## Abstract

When an excited electromagnetically open optical waveguide goes through a temporal transition of its material properties, it radiates to the ambient surroundings. In this paper, we explore this radiation and reveal, using asymptotic evaluation of path integral in the complex frequency (Laplace) plane, a peculiar space-time dependence of its frequency. Specifically, we derive an asymptotically exact formula (Eq. (11)) for the instantaneous radiation frequency, which exhibits a chirp behavior with respect to time. This simple formula depends on the ambient properties and on the longitudinal wavenumber *β* of the guided mode before the temporal transition but not on the specific waveguide structure or materials. In addition, we derive a *t* ^−3/2^ decay rate of the radiative field amplitude on time. We verify our analytic results using full-wave simulations of a dispersive and lossy indium tin oxide waveguide that undergoes smooth temporal long transitions over up to ∼200 cycles at the initially guided mode frequency. Thus, these theoretical findings offer valuable insights into the behavior of general optical waveguides experiencing temporal transitions and provide a powerful tool for analyzing and designing such THz and optical setups, with potential use in sensing and imaging.

## 1 Introduction

Temporal interfaces have been extensively investigated since late 1950s [1], [2], [3], [4], in the context of magnetoelastic waves [5], [6], in transmission line systems [7], [8], for signal manipulations, such as stretching, compression, and time-inversion [6], [7], [8], and in plasma [9], [10]. In recent years, the field has experienced a resurgence of interest [11], [12], [13], [14], [15], [16] due to extensive research on wave manipulations by metamaterials and advancements in technology, enabling practical high-rate temporal modulations (e.g., see [17], [18]). The integration of temporal variation of the guiding medium, often referred to as the “fourth dimension” for wave design [19], has garnered significant attention. This novel approach has shown promise in surpassing known performance bounds and revealing intriguing wave phenomena, and functionalities, such as inverse prism [20], impedance matching and transformers [21], [22], [23], [24], [25], absorption [26], [27], spatial filtering [28], [29], and temporal photonic crystals [30], for topological effects [31], amplified emission and lasing [32], phase conjugation in leaky wave antennas [33], as well as for synthetic axion response [34], for non-reciprocity [35], [36], as well as for double-slit time diffraction [37], time refraction [38], [39], Doppler shift [40], as well as second harmonic generation [41].

While temporal interfaces have been extensively studied theoretically, experimental demonstrations are less common. Implementing abrupt changes in the medium parameters on a large scale poses significant challenges. However, practical implementations of temporal discontinuities appear more feasible when confined to compact domains such as cavities [35], [36], [42], [43], [44], metasurfaces [45], [46], [47], [48], [49], [50], [51], [52], thin dielectric layers [26], [53], [54], and transmission lines [7], [8], [12], [21], [43], [55]. For instance, the authors in [8], [55] demonstrated experimentally signal compression and inversion by temporal discontinuities in transmission lines at radio frequencies, demonstrating effects such as signal stretching and compression, and time-reversal. In the THz and optical frequencies, transmission lines are impractical, and instead, thin dielectric layers and interfaces acting as waveguides can be used [18], [53], [54]. For instance, Bohm et al. [18] demonstrated optical switching of epsilon-near-zero plasmon resonances in indium tin oxide (ITO) layer, and, Miyamaru et al. [53], [54] experimentally demonstrated ultrafast frequency shifts in the THz range caused by a time-varying dielectric layer. Thus, providing the first steps towards practical potential implementations for temporal discontinuities in the optical regime.

In this paper, we focus on the radiation dynamics by a temporal transition of an optical waveguide. This problem is open and non-resonant by its nature and therefore challenging to be formulated using approximated techniques such as coupled mode theory [56], [57], [58], on the other hand, since the problem is time-varying, and the radiation effect is essentially a transient, it is less natural to be captured using a mode-matching approach that tailors a steady state solution composed of guided modes before the temporal transition with those supported following the transition [48], [52], [59], [60]. Transient dynamics can be captured using direct time-domain techniques [61], [62], alternatively compact and physically transparent solution to the this problem can be obtained using Laplace transform which is a highly fitted tool to accommodate initial value problems, and have implemented in temporally switched wave systems before [13], [49], [50], [63]. By solving the problem using this approach, we end up with a formally exact solution in the form of an inverse Laplace transform integral. However, recall that our main objective in this work is to explore the radiation emitted by the temporal transition. This is essentially a transient phenomenon, which we seek to describe in closed form. To that end, we apply asymptotic evaluation of complex path integrals about the branch cut singularity of a spectral integral that represents the radiation dynamics. This procedure yields an asymptotic closed-form formula for the *radiation wave* caused by the temporal transition. Intriguingly, we show that the radiation frequency possesses a chirp-like dependence on time. We show that this dependence is universal, independent of the specific waveguide geometry and materials, but only depends on the initial mode longitudinal wavenumber and on the ambient wave velocity.

## 2 Case study: temporally switched dielectric waveguide

**Figure 1:**
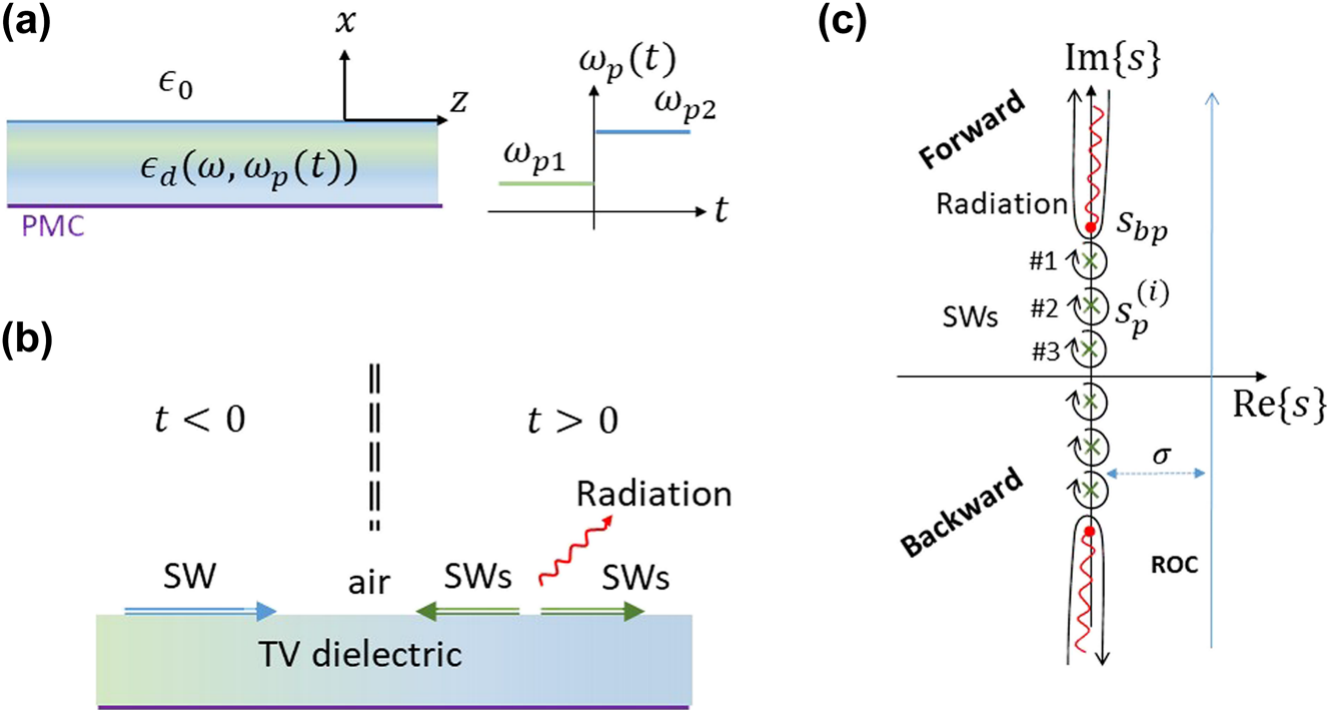
The studied space and spectral domain configuration. (a) Time-varying dielectric slab optical waveguide. The waveguide is surrounded by air with *ɛ* ≈ *ɛ*_0_ while the slab layer consists of a lossy dispersive time-varying dielectric material that its plasma frequency changes between two values, *ω*_*p*1_ and *ω*_*p*2_ at time *t* = 0. A perfect magnetic conductor backs the layer. (b) At *t* < 0 a TE surface wave (SW guided mode) is propagating. At *t* > 0, after the temporal discontinuity, forward and backward SWs, as well as radiation, are expected. (c) The complex *s* plane. Poles correspond to guided modes that may be excited after the switching, while the branch cuts correspond to radiation waves.

Inspired by the experimental setups in [18], [53], [54], we choose to examine the configuration depicted in Figure 1(a). We assume that the waveguide consists of a general dispersive dielectric that follows a typical Drude model with $\varepsilon_{d_{1}}=\varepsilon_{\infty 1}-\omega_{p1}^{2}/\left(\omega^{2}-j\omega \Gamma_{1}\right)$ where *ɛ*_∞1_, *ω*_*p*1_ and Γ_1_ denote the model parameters, high-frequency permittivity, plasma frequency, and damping rate, respectively. The waveguide thickness is *d* and is backed by a perfect magnetic conductor (PMC), implying that in a realistic setup, the system is assumed symmetric. This specific choice does not limit the validity of our result regarding the temporal chirped dynamics of the radiation frequency, which apply to any planar waveguide. The waveguide supports a guided mode, propagating in the +*z* direction, with wavenumber *β*. Thus, the fields can be expressed by

(1) $$E_{1}^{a,d}\left(r,t\right)=e_{1}^{a,d}\left(x,t\right)e^{-j\beta z},H_{1}^{a,d}\left(r,t\right)=h_{1}^{a,d}\left(x,t\right)e^{-j\beta z}$$

where superscript *a*, *d* stand for ‘air’ and ‘dielectric’. This initial field is time-harmonic with time-dependence $e^{j\omega_{0}t}$ . At *t* = 0 an abrupt temporal transition of the dielectric material properties occures (smooth transitions are considered later). At *t* > 0 we assume to have a new plasma frequency in the Drude model: *ω*_*p*2_, while the remaining parameters are not altered Γ_2_ = Γ_1_ and *ɛ*_∞2_ = *ɛ*_∞1_. Consequently, the fields are altered while the electric and magnetic fluxes remain continuous. We denote the fields at *t* > 0 by $E_{2}^{a,d}\left(r,t\right)$ and $H_{2}^{a,d}\left(r,t\right)$ . Note that the new fields are not time-harmonic anymore. Instead, they consist of various wave components, guided modes, and radiation waves with different time dependencies and frequencies (see illustration in Figure 1(b)). In order to find the fields after the temporal discontinuity, we apply the unilateral Laplace transform, as previously used e.g., in [13], [49], [50], [63]. The detailed derivation for our problem is provided in the Supplementary Material file. Briefly, since the waveguide dielectric is assumed dispersive, we have to impose the following continuity across the temporal boundary at *t* = 0 to obtain the fields at *t* = 0^+^,

(2) $$Y_{1}^{a,d}\left(r,t^{0-}\right)=Y_{2}^{a,d}\left(r,t^{0+}\right),$$

where, for non-magnetic dielectric material ***Y*** stands for the electric field ***E***, magnetic induction ***B***, polarization density ***P*** and its time-derivative ∂_*t*_ ***P*** in the air domain and in the dielectric slab. Eq. (2) enforces that the *z* behavior of the fields is preserved (conservation of quasi-momentum in *z*) through the temporal boundary, thus implying that the *z* dependence of $E_{2}^{a,d}$ and $H_{2}^{a,d}$ is also e^−j*βz*^ and as a result *∂*_*z*_ → −*jβ* in Maxwell’s equations (Supplementary Material) (Section S1.2 Eq. (S6)). The fields at *t* = 0^+^, which are obtained by the continuity conditions in Eq. (2), serve as spatial initial conditions for a solution of the Helmholtz equation formulated in the Laplace domain for the wave-fields amplitude at *t* > 0. The latter reads,

(3) $$\partial_{x}^{2}{\tilde{h}}_{z}\left(x,s\right)-\gamma^{2}{\tilde{h}}_{z}\left(x,s\right)=F\left(x,t=0^{+};s\right),$$

where ${\tilde{h}}_{z}={\tilde{h}}_{z}^{\left(a,d\right)}$ , and $\gamma =\gamma_{\left(a,d\right)}$ . The right-hand side of Eq. (3) is given explicitly in the Supplementary Material file (see Eqs. (S10) and (S37) there), together with *γ*_*d*_, which possesses a relatively complex mathematical structure (Eqs. (S15) and (S46) in the Supplementary Material). Since we are interested in the radiation into the air domain, the main focus of the discussion is *γ* = *γ*_*a*_ that reads,

(4) $$\gamma_{a}=\sqrt{s^{2}/c^{2}+\beta^{2}}$$

and *c* is the speed of light in a vacuum (used for the air domain). The solution of ${\tilde{h}}_{z}\left(x,s\right)$ in Eq. (3) is provided in Eqs. (S47)–(S48) in Supplementary Material. Next, the inverse Laplace transform is applied to obtain the time domain fields,

(5) $$h_{z}^{a,d}\left(x,t\right)=\frac{1}{2\pi j}\overset{j\infty +\sigma }{\underset{-j\infty +\sigma }{\int }}{\tilde{h}}_{z}^{a,d}\left(x;s\right)e^{st}ds.$$

The physical realization of the switching process dictates that following the abrupt switching of the slab’s dielectric constant, a set of orthogonal surface wave modes of the medium are excited in addition to transient radiation (continuous spectrum). This is readily observed in the Laplace formulation. Specifically, the spectral function ${\tilde{h}}_{z}^{a,d}\left(x;s\right)$ contains two types of singularities; isolated poles, and branch points with their corresponding branch-cuts (for further details, refer to Section S1.4 in Supplementary Material). See Figure 1(c) for illustration. Formally, the integration path parameter *σ* > 0 should be selected such that the integration is performed in the transform region of convergence, namely, since our problem is causal and stable, to the right of the imaginary axis on the *s*-plane. However, to calculate the fields at *t* > 0 this formal integration path can be deformed to encircle the singular points in the complex *s* plane. Thus, we get deeper physical insight on the various wave species that are excited during the temporal discontinuity. Specifically, the pole singularities, coming in conjugate pairs, correspond to the counter-propagating guided modes in the ±*z* directions after the temporal switching. Moreover, besides the poles, there are two additional branch point singularities that originate from the square roots in the complex propagation constant along the *x* direction, in the air, and in the dielectric, *γ*_*a*_ and *γ*_*d*_, respectively. However, it can be verified that ${\tilde{h}}_{z}\left(x,s\right)$ is even in *γ*_*d*_, and therefore *γ*_*d*_ = 0 is not a branch point of the spectral field (see discussion following Eq. (S51) in Supplementary Material). This leaves only the branch points due to *γ*_*a*_. The integration around the branch point provides a continuous frequency spectrum of waves that are excited due to the temporal discontinuity and radiated into the air domain. Their behavior is elaborated below and is the key result of this paper.

## 3 Derivation of the radiative field

Our analysis begins by writing the integral around the branch cut with branch point at *s*_*bp*_ (upper branch in Figure 1(c)). The integration takes the following canonical form

(6) $$I_{bc}^{U}=\underset{bc}{\int }f\left(s\right){\text{e}}^{\psi \left(s\right)}ds,$$

where $f\left(s\right)$ is a slow varying function in *s*. For example for the *E*_*y*_ field component it reads,

$$f\left(s\right)=\frac{1}{2\pi j}\frac{\mu_{0}sD\left(s\right)}{\gamma_{a}\left(s\right)}$$

and it depends on the field’s initial conditions as well as on the boundary and the time continuity conditions (see Eqs. (S19) and (S49) in Supplementary Material for the expression of $D\left(s\right)$ ). Furthermore, $\psi \left(s\right)$ in Eq. (6) is given by

(7) $$\psi \left(s\right)=-\gamma_{a}\left(x-d\right)+st$$

denoting the phase term. As opposed to $f\left(s\right)$ which is slowly varying and independent of the physical coordinates *x* and *t*, the exponential term in Eq. (6) is highly oscillatory except in the vicinity of a stationary point, if exists. The integration in Eq. (6) is carried around the upper branch cut as shown in Figure 1(c). In this case, the variable *s* passes in the range $\left(i\infty +\varepsilon ,s_{bp}+\varepsilon \right)\cup \left(s_{bp}-\varepsilon ,i\infty -\varepsilon \right)$ where *ɛ* → 0^+^. Along this *s*-path, the variable *γ*_*a*_ maps into the more convenient range $\left(i\infty ,-i\infty \right)$ . Therefore, we perform the following change of integration variable: *s* → *γ*_*a*_. Recall Eq. (4), from which we have d*s* = d*γ*_*a*_ *c* ^2^ *γ*_*a*_/*s*. By plugging the above change of variables into Eq. (6) we get,

(8) $$I_{bc}^{U}=\overset{-i\infty }{\underset{i\infty }{\int }}\frac{c^{2}\gamma_{a}}{s\left(\gamma_{a}\right)}f\left(s\left(\gamma_{a}\right)\right){\text{e}}^{\psi \left(s\left(\gamma_{a}\right)\right)}d\gamma_{a}.$$

**Figure 2:**
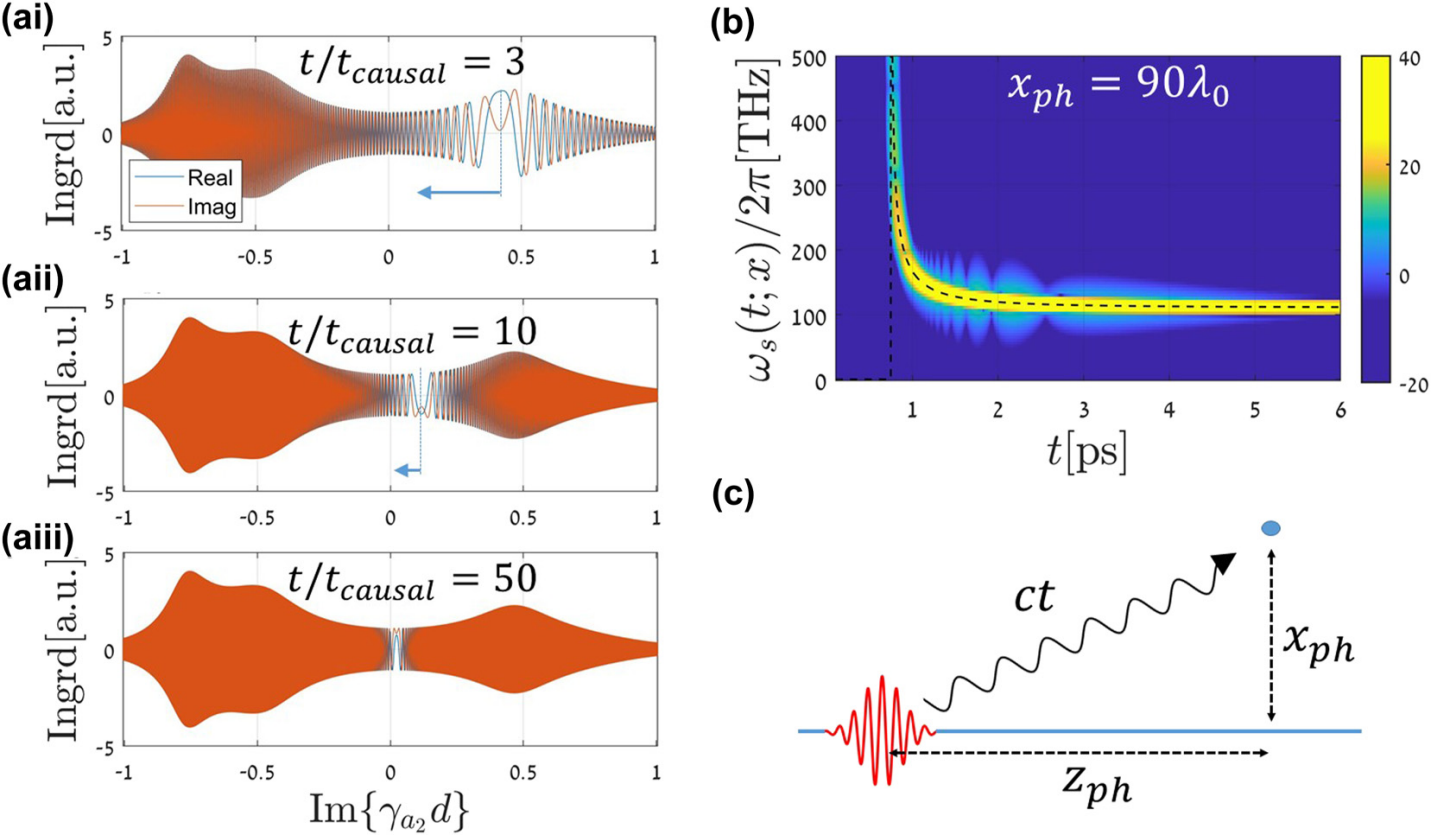
The observed radiation frequency. (ai) Numerical demonstration of the stationary phase for a fixed distance *x*_*ph*_ = 90*λ*_0_ from the interface, and at a fixed time *t* = 3*t*_causal_, not too long after the causal time *t*_causal_ = *x*_*ph*_/*c*. (aii, aiii) As (ai) but for a longer time after the temporal switching, *t* = 10*t*_causal_, and *t* = 50*t*_causal_. The stationary point is more localized and gets closer to the branch point at *γ*_*a*_ = 0 as the time elapses. (b) A spectrogram picture, frequency versus time, for an observer located at *x*_*ph*_ = 90*λ*_0_. The dominant frequency varies with time and precisely follows the asymptotic formula for the radiation frequency in Eq. (10). (c) A “quantum mechanical” interpretation for the stationary phase result. The photon here is treated as a particle.

For a remote observer, *x* ≫ *λ* and long enough time after the causal time at which the wavefront reaches the observer at *x*, the integrand in Eq. (8) possesses a stationary phase point behavior as shown in Figure 2(ai)–(aiii). The stationary phase point is found by solving $\psi^{\prime }\left(\gamma_{a}\right)=0$ , which leads to

(9) $$\gamma_{a,s}=\frac{\beta \left(x-d\right)}{\sqrt{{\left(x-d\right)}^{2}-{\left(ct\right)}^{2}}}.$$

This stationary point is imaginary and thus located along the integration path only in causal times, i.e., for *ct* > *x* − *d*. Asymptotically, the contribution to the branch cut integral is, therefore, dominated by the stationary point contribution and takes the following form [64], Ch. 4],

(10) $$\begin{array}{ll} I_{bc} & ∼\sqrt{-j2\pi \frac{\omega_{s}^{3}\left(x,t\right)}{\omega_{bp}^{2}t}}\frac{c^{2}\gamma_{a,s}}{s\left(\gamma_{a,s}\right)}f\left(s\left(\gamma_{a,s}\right)\right)\\ & \;\times {\text{e}}^{\psi \left(s\left(\gamma_{a,s}\right)\right)}H\left(t-\frac{x-d}{c}\right), \end{array}$$

where $H\left(\cdot \right)$ denotes the Heaviside step function, representing the fact that the wavefront reaches the observer at the casual time $t_{\text{causal}}=\left(x-d\right)/c$ , and $\omega_{s}\left(x,t\right)$ as well as *ω*_*bp*_ are given in Eq. (11) below. Using Eq. (9), it is straightforward to show by Eq. (10) that the radiative field amplitude exhibits an asymptotic time dependence ∼*t* ^−3/2^ for a fixed observer location *x*.

### 3.1 Chirped-like radiation frequency

In order to identify the radiation frequency due to the temporal transition we resort back into the *s* variable that is directly connected to the temporal frequency. Using Eqs. (4) with (9) we have

(11) $$\omega_{s}\left(x,t\right)=-js\left(\gamma_{a,s}\right)=\frac{\beta c^{2}t}{\sqrt{{\left(ct\right)}^{2}-{\left(x-d\right)}^{2}}}.$$

Equation (11) is the main result of this paper. It implies that the radiation frequency depends on space and time and exhibits a chirp-like *behavior*. For a fixed observer location *x*, and a long time after the wavefront arrival time (*t*_casual_), the radiation frequency settles at *ω*_*s*_ → *βc* = −*js*_*bp*_ = *ω*_*bp*_. However, shortly after the wavefront arrival time, the frequency exhibits a singular behavior as the denominator in Eq. (11) vanishes. Mathematically, this result results from the stationary phase point motion as time progresses. This behavior is shown in Figure 2(ai)–(aiii) for three time instances, $t=\left(3,10,50\right)t_{\text{causal}}$ , respectively. All the numerical results in this paper are shown for a layer of thickness *d* = 0.2*λ*_0_, where *λ*_0_ = 2*πc*/*ω*_0_ where $\omega_{0}=2\pi \cdot 120\left[\text{rad}\cdot \text{THz}\right]$ is the excitation frequency of the guided modes prior to the temporal transition, and with Drude model parameters: $\omega_{p1}=1376\left[\text{rad}\cdot \text{THz}\right],\Gamma_{1}=147\left[\text{rad}\cdot \text{THz}\right],\varepsilon_{\infty 1}=4.31$ (the model values were taken from [65]) before the transition, and a 15 % higher plasma frequency $\omega_{p2}=1582\left[\text{rad}\cdot \text{THz}\right]$ following the transition. The resulting time-frequency behavior is shown in a spectrogram given in Figure 2(b). The spectrogram that is calculated for the exact solution is compared with the curve obtained by the asymptotic result in Eq. (11) (dashed black line). An excellent agreement between the results is evident. Remarkably, as will be shown below, using finite-difference-time-domain (FDTD) simulations, this chirp behavior also holds nicely for gradual, highly smooth temporal transitions.

### 3.2 “Quantum mechanical” interpretation of the radiation frequency

The derivation of Eq. (11) above required extensive analytical work. Here, using simple kinematic arguments we provide an alternative derivation, that while not strictly formal, provides some physical insight into the peculiar time-frequency behavior of Eq. (11). To that end, consider a radiative photon emitted at the temporal switching time *t* = 0, from a point −*z*_*ph*_ along the interface. We denote by *t* the time it propagates before reaching the observer at *z* = 0 and *x*_*ph*_ = *x* − *d*. Thus, the photon passes along a distance equals to *ct*, which means that it passes along parallel to the interface distance that equals to $z_{ph}={\left({\left(ct\right)}^{2}-x_{ph}^{2}\right)}^{0.5}$ . See Figure 2(c) for illustration. On the other hand, along the *z* direction, we have conservation of momentum during the switching process. Using de-Broglie hypothesis, the photon quasi-momentum component along *z* equals *p*_*z*,*ph*_ = *βℏ*, while the photon effective mass reads *m*_eff_ = *E*/*c* ^2^ = *ℏω*/*c* ^2^. Thus,

(12) $$z_{ph}=v_{z,ph}t=\frac{p_{z,ph}}{m_{\text{eff}}}t.$$

By plugging the expressions for the quasi-momentum and the effective mass, we immediately obtain Eq. (11) above. Obviously, this quantum-like derivation should be demystified by noting that the definition of a photon mass here should be considered only as an elegant manifestation of the phase velocity, and its connection to that of the guided mode before the temporal switching. Yet, this simplistic derivation captures nicely complicated wave physics.

### 3.3 Universality of the radiation characteristics

The slab material can be characterized using different models that are applicable to various microscopic material pictures [66], [67], [68], [69]. These, in turn, may also imply variation in the required continuity conditions that are applied given that different model parameters are to be switched at the transition time *t* = 0. The specific choice of the slab material model will determine the specific amplitude of the radiated field, as encapsulated in the function $f\left(s\left(\gamma_{a,s}\right)\right)$ given in Eq. (10). In contrast, however, the field amplitude decay rate as *t* ^−3/2^ is a universal trend that results from the structure of the *phase term* $\psi \left(s\right)$ in the spectral integral (see Eq. (10)). This is since asymptotically, $I_{bc}\propto 1/\sqrt{\left|\psi {\left(s\left(\gamma_{a}\right)\right)}^{\prime \prime }\right|}$ at *γ*_*a*_ = *γ*_*a*,*s*_, and $\psi \left(s\left(\gamma_{a,s}\right)\right)$ depends on the observer location *x*, the time elapsed *t*, as well as on the speed of light *c* in the ambient surrounding and on the longitudinal wavenumber of the guided mode *β* prior the temporal transition.

Using the same argument, and even more interestingly, the specific chirped frequency-time dependence of the radiated wave is entirely independent of the material model of the slab. This point, besides the mathematical argument discussed above, can be intuitively understood using the kinematic momentum/energy conservation argument used in Section 3.2.

## 4 Gradual temporal transitions

**Figure 3:**
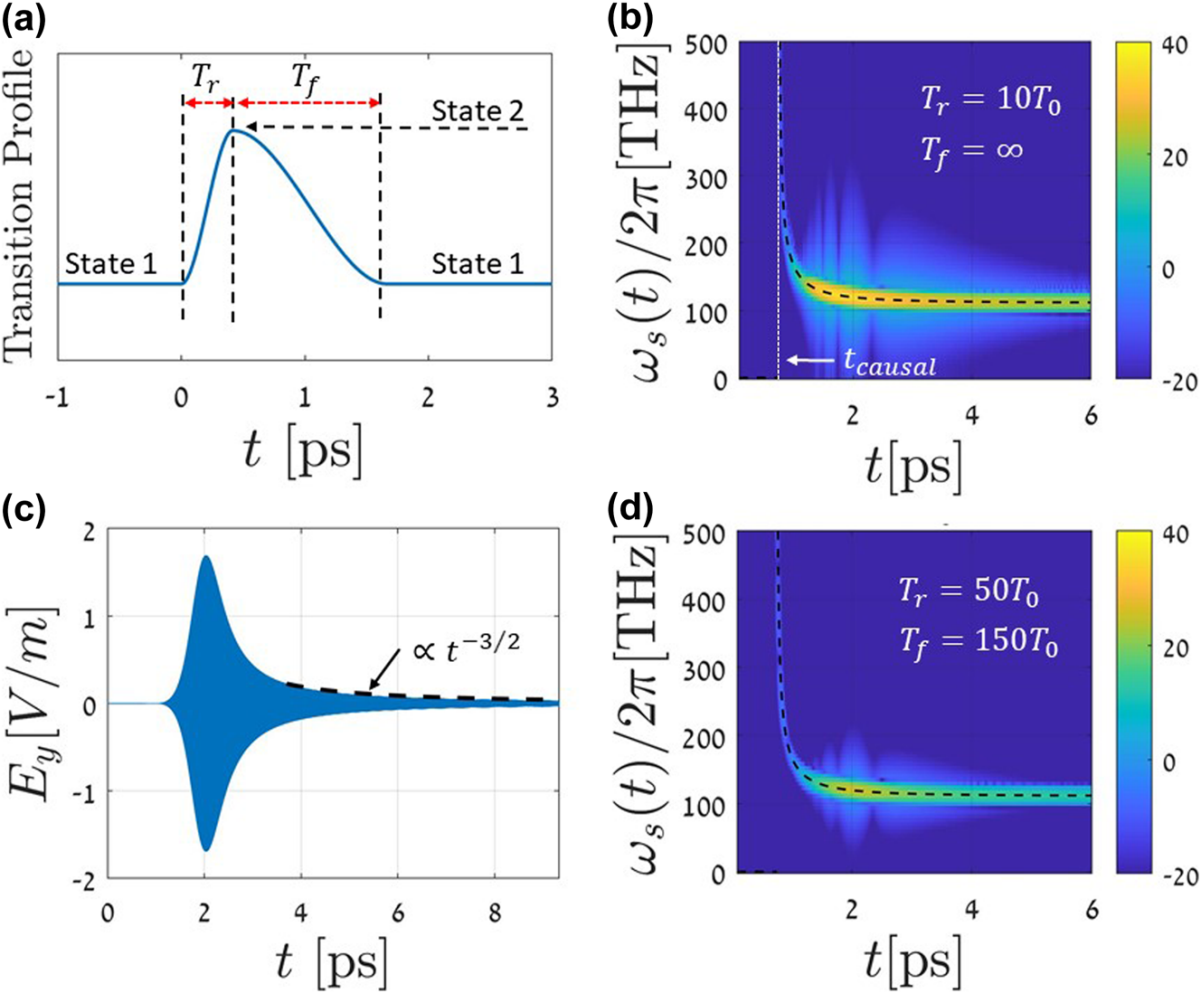
Soft transitions studied by FDTD simulations and compared to the analytical model. (a) The transition profile of the plasma frequency. (b) Spectrogram of the FDTD simulation field at *x* = 90*λ*_0_ with transition rise time *T*_*r*_ = 10*T*_0_ and infinite fall time (i.e., a transition from state 1 to state 2). The wavefront arrival time is marked by *t*_causal_. The analytic result in Eq. (11) is shown by the black dashed line, indicating in excellent agreement with the gradual transition case. (c) and (d) Simulation results with an even slower transition *T*_*r*_ = 50*T*_0_ and with relaxation *T*_*f*_ = 150*T*_0_ (state 1 to 2 and back to 1 – see (a)). The observer is located at *x* = 90*λ*_0_. (c) The electric field at *x* = 90*λ*_0_. The field oscillations are too dense to be discerned in this scale. The envelope asymptotically decays as *t* ^−3/2^. (d) As (b) but for the transition scenario in (c).

In this section, we demonstrate by full-wave FDTD simulations (using in-house developed code, using [70]) that the theoretical prediction of the radiation frequency in Eq. (11) also holds for the case of gradual temporal transitions. In Figure 3(a), we provide the transition profile. This profile is applied to the plasma frequency. Thus, we have in our FDTD simulations $\omega_{p}\left(t\right)$ – that is varying continuously in time. For simplification, we assume that the other parameters of the Drude model, Γ and *ɛ*_∞_, remain unchanged. The profile function in Figure 3(a) represents the transition between the two material states, 1 and 2. In the general case, the transition process involves a rise time *T*_*r*_ from state 1 with *ω*_*p*1_ to state 2 with *ω*_*p*2_, followed by a slower relaxation process with a fall-time *T*_*f*_ from state 2 back to 1. The Drude model parameters used in these simulations are identical to those used to calculate Figure 2. In Figure 3(b), only a single transition occurs from state 1 to state 2. The rise time is taken to be *T*_*r*_ = 10*T*_0_ when *T*_0_ denotes the period of the guided mode before the transition starts; thus, $T_{0}=2\pi /\omega_{0}=8.33\;\left[\text{fs}\right]$ . In this example, we consider no relaxation, and thus, *T*_*f*_ = ∞. The field spectrogram at *x* = 90*λ*_0_ shows the chirped radiation frequency behavior with an excellent agreement to the formula in Eq. (11). As expected, the wavefront reaches the observer at the causal time. At that instant, the radiation frequency is very high and gradually decreases over time to the steady radiation frequency corresponding to the branch point. Figure 3(c) and (d) show results with a more realistic transition involving a slower temporal transition, $T_{r}=50T_{0}=0.42\;\left[\text{ps}\right]$ , and relaxation time *T*_*f*_ = 150*T*_0_, thus a total transition duration of about 200 cycles. The time axis of Figure 3(a) corresponds quantitatively to this case. In Figure 3(c) the electric field at *x* = 90*λ*_0_ is observed, clearly demonstrating the theoretically anticipated *t* ^−3/2^ decay of field amplitude. In Figure 3(d) a spectrogram of the field (as in Figure 3(b)) is shown, and an excellent agreement is obtained by comparison to the theoretically anticipated frequency-time dependence in Eq. (11) that is shown by the black-dashed curve.

## 5 Conclusions

This paper explores the radiation dynamics due to temporal transition in an electromagnetically open waveguide. Our approach provides a comprehensive understanding of the wave dynamics during the temporal switching. In particular, our approach sheds light on the unique radiation characteristics, exhibiting universal radiation frequency behavior independent of the specific waveguide configuration as given by Eq. (11) above, and with an asymptotic ***t*** ^−**3/2**^ decay rate of the field amplitude. These results hold firmly also in the case of smooth temporal transitions as validated for realistic gradual transitions in Figure 3 with switching duration as slow as ∼200 cycles of the original wave. As such, our quantitative description of the radiation dynamics can be used as a unique fingerprint applied in far-field sensing and characterization of temporal transitions in nanoscale THz and optical devices, as well as a new venue to explore optical non-equilibrium processes as studied in [71].

## Supplementary Material

Supplementary Material Details
