# Supplementary material for: Universal transient radiation dynamics by abrupt and soft temporal transitions in optical waveguides
Source: Nanophotonics. 2025 Jan 6;14(6):785–93. doi: 10.1515/nanoph-2024-0525 (PMC11964138; doi:10.1515/nanoph-2024-0525)
Supplement: Supplementary file 1 — Supplementary Material Details [file j_nanoph-2024-0525_suppl_001.pdf]

Universal transient radiation dynamics by abrupt and soft temporal transitions in optical waveguides

Amir Shlivinski
School of Electrical and Computer Engineering,
Ben Gurion University of the Negev, Beer Sheva, Israel, 84105

Yakir Hadad
School of Electrical Engineering,
Tel-Aviv University, Ramat-Aviv, Tel-Aviv, Israel, 69978

December 18, 2024

Abstract

This part of the paper provides the mathematical derivations of the expressions that are discussed in the main body of text. The main text contains results derived for a *dispersive and lossy* dielectric waveguide. The detailed derivation for this case is provided in Sec. 2 below. For didactic reasons, in Sec. 1 we begin by analyzing the simpler problem of dispersion-less dielectric waveguide. This can assist in reading Sec. 2 since it involves essentially the same mathematical steps, but with substantially simpler expressions. In addition, in order to assist the reader, in Fig. S1 below we provide a sketch of the mathematical formulation of the solution we apply. It is described in detail in the following subsections.

Contents

1 Detailed solution for dispersion-less dielectric slab problem **2**

1.1 Description of the initial settings 2

1.2 Continuity conditions after the time switching at $t = 0$ 3

1.3 Formulation of the initial value problem for the fields after $t = 0^+$ 4

1.4 Singular points 7

2 Dispersive and Lossy Plasmonic slab **9**

2.1 Singular points 13

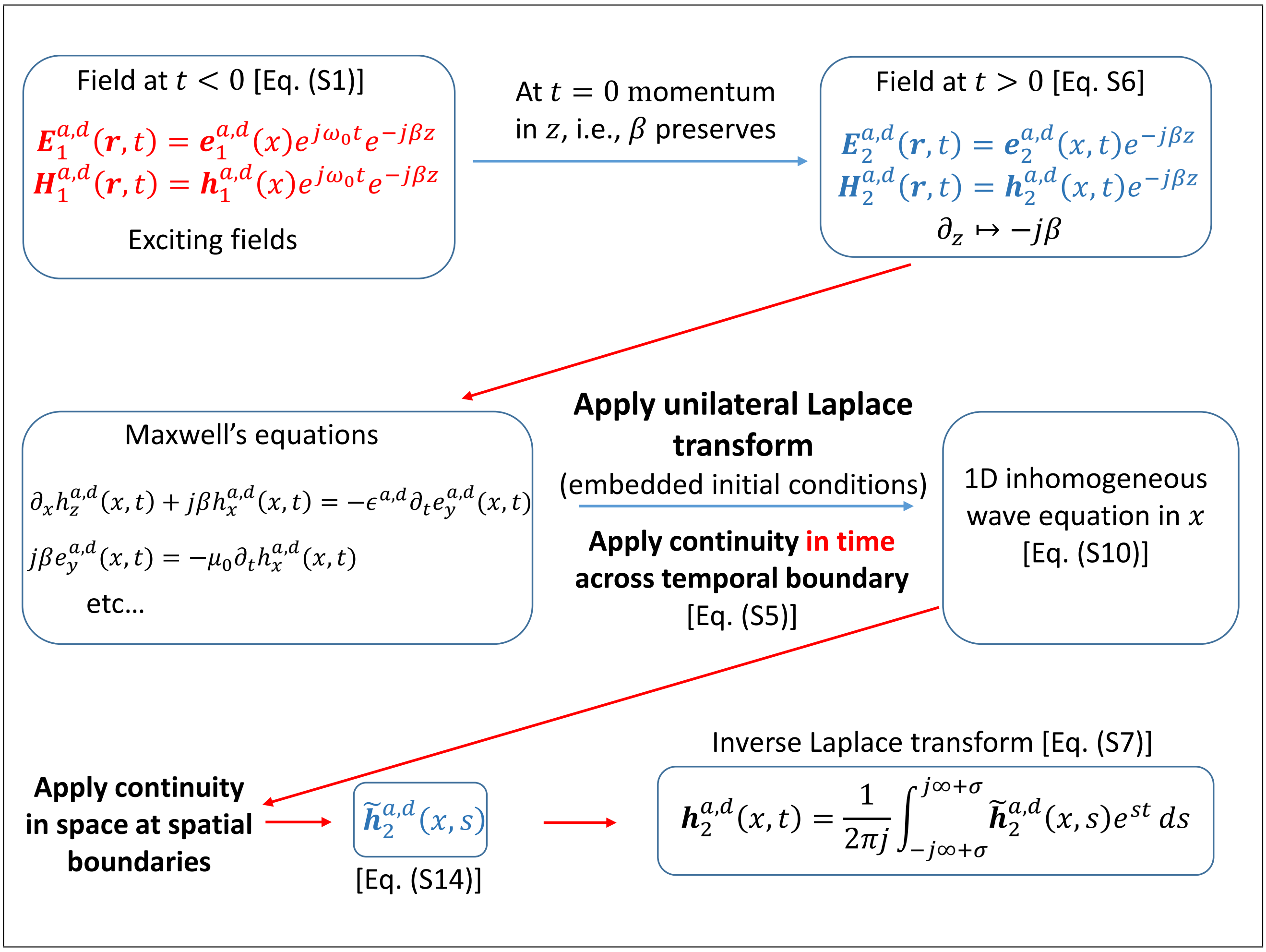

Figure S1: A scheme for the solution of the optical waveguide temporal switching problem using the unilateral Laplace transform.

1 Detailed solution for dispersion-less dielectric slab problem

1.1 Description of the initial settings

Initially at $t < 0$, h and k_c are the transversal wave numbers for $x > d$ and $x < d$ respectively, d is the slab's thickness, $\epsilon_{d_{r_1}}$ is it's relative permittivity, β is the longitudinal wave number along z and ω_0 is the radian frequency. The medium above the slab at $x > d$ is air (considered as vacuum) with ϵ_{r_a}. The slab is backed by a perfect magnetic conductor (PMC) at $x = 0$. Thus, this structure is equivalent by mirror symmetry to a symmetric slab supporting even modes. Assuming a TE surface mode propagating in the z direction with the electromagnetic fields:

$$\boldsymbol{H}_1(x,z,t) = \boldsymbol{h}_1(x)e^{-j\beta z}e^{-j\omega_0 t} \tag{S1a}$$

$$\boldsymbol{E}_1(x,z,t) = \boldsymbol{e}_1(x)e^{-j\beta z}e^{-j\omega_0 t} \tag{S1b}$$

where $\boldsymbol{h}_1(x) = [\hat{\boldsymbol{x}}h_x(x,y) + \hat{\boldsymbol{z}}h_z(x,y)]$ and $\boldsymbol{e}_1(x) = \hat{\boldsymbol{y}}e_y(x,y)$ and with

$$h_z(x,y) = \begin{cases} \sin(k_c x) & \text{for } 0 \leq x \leq d \\ \\ e^{-h(x-d)}\sin(k_x d) & \text{for } d \leq x \end{cases} \tag{S2a}$$

$$h_x(x,y) = \begin{cases} ,\ \frac{-j\beta}{k_c}\cos(k_c x) & \text{for } 0 \leq x \leq d \\ \\ \frac{-j\beta}{h}e^{-h(x-d)}\sin(k_x d) & \text{for } d \leq x \end{cases}, \tag{S2b}$$

$$e_y(x,y) = \begin{cases} \frac{j\omega\mu}{k_c}\cos(k_c x) & \text{for } 0 \leq x \leq d \\ \\ \frac{j\omega\mu}{h}e^{-h(x-d)}\sin(k_x d) & \text{for } d \leq x \end{cases}, \tag{S2c}$$

where k_c and h are the transversal to z wavenumbers in the slab, and β is the longitudinal wavenumber. Following the formulation in [1], the dispersion relation is:

$$k_c \tan(k_c d) = h, \tag{S3}$$

with

$$k_0^2 \epsilon_{a_r} = \beta^2 - h^2, \tag{S4a}$$

$$k_0^2 \epsilon_{d_{r_1}} = \beta^2 + k_c^2. \tag{S4b}$$

Solving (S4) with (S3) gives the set of wavenumbers for surface wave modal propagation.

1.2 Continuity conditions after the time switching at $t = 0$

At time $t = 0$ the slab's permittivity is switched from $\epsilon_{d_{r_1}}$ to $\epsilon_{d_{r_2}}$ while the surrounding medium ($x > d$) remains unchanged with ϵ_{a_r}. Since the medium is non-magnetic with consrtant permeability, μ_0, continuity of the magnetic flux implies the continuity of the magnetic field. Thus at $t = 0$ we have

$$\boldsymbol{H}_1^{(a,d)}(x,z,0^-) = \boldsymbol{H}_2^{(a,d)}(x,z,0^+). \tag{S5a}$$

Since in this section the slab is non-dispersive it is enough to require the continuity of the electric displacement, $\boldsymbol{D}$, with no need to resort for the polarization and its time derivative. Thus,

$$\epsilon_{d_{r_1}}\boldsymbol{E}_1^{(d)}(x,z,0^-) = \epsilon_{d_{r_2}}\boldsymbol{E}_2^{(d)}(x,z,0^+), \qquad \epsilon_{a_r}\boldsymbol{E}_1^{(a)}(x,z,0^-)x = \epsilon_{a_r}\boldsymbol{E}_2^{(a)}(x,z,0^+). \tag{S5b}$$

Here and henceforth, we use subscript 1 and 2 in order to denote fields before and after the time switching, respectively. Also, we use the superscripts a and d in order to denote the fields in the dielectric and the air, respectively. As a result of Eqs. (S5), the spatial z dependence of the field has to remain unaltered by the temporal switching and consequently we have

$$\boldsymbol{H}_2(x,z,t) = \boldsymbol{h}_2(x,t)e^{-j\beta z} \tag{S6a}$$

$$\boldsymbol{E}_2(x,z,t) = \boldsymbol{e}_2(x,t)e^{-j\beta z} \tag{S6b}$$

Note in Eqs. (S6) that the field polarization remains TE as it was before the switching. Moreover, note that the time-dependence is not time-harmonic. Instead, it will contain various time dependencies for different waves that are excited by the time discontinuity. Therefore, it is now introduced inside the transverse field functions $\boldsymbol{e}_2(x,t)$ and $\boldsymbol{h}_2(x,t)$.

1.3 Formulation of the initial value problem for the fields after $t = 0^+$

The solution for the fields at $t > 0$ (after the temporal discontinuity) is carried out using the unilateral Laplace transform. In light of the discussion above regarding the z dependence, the fields can be expressed in the following form using the inverse Laplace transform,

$$H_z(x,z,t) = e^{-j\beta z}\frac{1}{2\pi j}\int_{-j\infty+0^+}^{j\infty+0^+} ds\,\widetilde{h}_z(x,s)e^{st} \tag{S7a}$$

$$E_y(x,z,t) = e^{-j\beta z}\frac{1}{2\pi j}\int_{-j\infty+0^+}^{j\infty+0^+} ds\,\widetilde{e}_y(x,s)e^{st} \tag{S7b}$$

and similarly for H_x. Over-tilde, $\widetilde{}$ denotes Laplace transformed quantities. Specifically, for a general function $f(x,t)$ the Laplace transform counterpart $\widetilde{f}(x;s)$ reads,

$$\widetilde{f}(x,s) = \mathcal{L}\{f(x,t\} = \int_0^\infty f(x,t)e^{-st}dt, \tag{S8}$$

where f denotes each one of the TE field components, h_x, h_z or e_y. Additionally, applying (S8) with the temporal derivatives of $f(x,t)$ gives [2],

$$\begin{aligned}
&\mathcal{L}\{\partial_t f(x,t\} = s\widetilde{f}(x,s) - f(x,t=0^+),\\
&\mathcal{L}\{\partial_t^2 f(x,t\} = s^2\widetilde{f}(x,s) - sf(x,t=0^+) - \partial_t f(x,t=0^+)
\end{aligned} \tag{S9}$$

where $\partial_t f(x,t)$ is the temporal derivative of $f(x,t)$. Obviously, for the solution ansatz in Eq. (S7), $\partial_z \mapsto -j\beta$. By using the latter with Eqs. (S9) in Maxwell's equations for the TE polarization, we readily obtain the following inhomogeneous 1D Helmholz equation for the partial field $\widetilde{h}_z(x,s)$

$$\partial_x^2 \widetilde{h}_z(x,s) - \left[\left(\frac{s}{v}\right)^2 + \beta^2\right] \widetilde{h}_z(x,s) = -\frac{1}{v^2}\left[s h_z(x,0^+) + \partial_t h_z(x,0^+)\right] \tag{S10}$$

where $\widetilde{h}_z = \widetilde{h}_z^{(a)}$ with $v = v^{(a)}$ for $x > d$ and $\widetilde{h}_z = \widetilde{h}_z^{(d)}$ with $v = v^{(d)}$ for $0 \le x \le d$ where

$$v^{(a)} = v_a = \frac{c}{\sqrt{\epsilon_{a_r}}}, \qquad v^{(d)} = v_{d_2} = \frac{c}{\sqrt{\epsilon_{d_{r_2}}}}, \tag{S11}$$

and with $c = 1/\sqrt{\mu_0\epsilon_0}$ denoting the speed of light in vacuum. In the right hand side of Eq. (S10), $h_z(x,0^+)$ and $\partial_t h_z(x,0^+)$ are the h_z-field and its temporal derivative at $t = 0^+$ following the switching of the dielectric constants of the medium. The $h_z(x,0^+)$ and $\partial_t h_z(x,0^+)$ are found using the continuity of the fluxes at $t = 0^\pm$ given in Eqs. (S5), with

$$\partial_t h_z^{(a)}(x,0^+) = -\frac{1}{\mu_0}\partial_x e_y^{(a)}(x,0^+) = -\frac{1}{\mu_0}\partial_x e_y^{(a)}(x,0^-) \tag{S12}$$

and

$$\partial_t h_z^{(d)}(x,0^+) = -\frac{1}{\mu_0}\partial_x e_y^{(d)}(x,0^+) = -\frac{1}{\mu_0}\frac{\epsilon_{d_{r_1}}}{\epsilon_{d_{r_2}}}\partial_x e_y^{(d)}(x,0^-) \tag{S13}$$

Using these expressions with those of Eq.(S2) in Eq.(S10), solving and satisfying the initial conditions at $t = 0^+$ gives,

$$\widetilde{h}_z(x,s) = \begin{cases} C\sinh(\gamma_{d_2}x) + \dfrac{s + j\omega_0\frac{\epsilon_{d_{r_1}}}{\epsilon_{d_{r_2}}}}{s^2 + \omega_0^2\frac{\epsilon_{d_{r_1}}}{\epsilon_{d_{r_2}}}}\sin(k_c x) & \text{for } 0 \le x \le d, \\ De^{-\gamma_a(x-d)} + \frac{s+j\omega_0}{s^2+\omega_0^2}e^{-h(x-d)}\sin(k_c d) & \text{for } d \le x, \end{cases} \tag{S14}$$

with C and D are s-dependent coefficients that will be determined, below, by imposing the continuity of the tangential fields at $x = d$ and

$$\gamma_a = \sqrt{\left(\frac{s}{v_a}\right)^2 + \beta^2}, \qquad \gamma_{d_2} = \sqrt{\left(\frac{s}{v_{d_2}}\right)^2 + \beta^2}, \qquad \mathrm{Re}\{\gamma_a, \gamma_{d_2}\} > 0. \tag{S15}$$

Once $\widetilde{h}_z(x,s)$ was obtained, the expressions for $\widetilde{e}_y(x,s)$ and $\widetilde{h}_x(x,s)$ can be obtained by applying the

Laplace transform to Maxwell's equations to obtain the following system of equations

$$\begin{bmatrix} s\epsilon_{a,d} & j\beta \\ j\beta & s\mu_0 \end{bmatrix} \begin{bmatrix} \widetilde{e}_y^{(a,d)}(x,s) \\ \widetilde{h}_x^{(a,d)}(x,s) \end{bmatrix} = \begin{bmatrix} e_y^{(a,d)}(x,0^+) - \partial_x \widetilde{h}_z(x,s) \\ \mu_0 h_x^{(a,d)}(x,0^+) \end{bmatrix}. \tag{S16}$$

Specifically, for $0 \leq x \leq d$

$$\widetilde{e}_y(x,s) = \frac{-\mu_0}{\gamma_{d_2}^2}\left[sk_c \frac{s + j\omega_0 \frac{\epsilon_{d_{r_1}}}{\epsilon_{d_{r_2}}}}{s^2 + \omega_0^2 \frac{\epsilon_{d_{r_1}}}{\epsilon_{d_{r_2}}}} + \frac{1}{k_c}\left(\beta^2 - j\omega_0 \frac{\epsilon_{d_{r_1}}}{\epsilon_{d_{r_2}}}\frac{s}{v_{d_2}^2}\right)\right]\cos(k_c x) - \mu_0 s C \frac{\cosh(\gamma_{d_2} x)}{\gamma_{d_2}} \tag{S17a}$$

and for $x > d$

$$\widetilde{e}_y(x,s) = \frac{-\mu_0}{\gamma_a^2}\left[\frac{1}{k_c}\left(\beta^2 - j\omega_0 \frac{s}{v_a^2}\right)\cos(k_c d) - sh\sin(k_c d)\frac{s + j\omega_0}{s^2 + \omega_0^2}\right] e^{-h(x-d)} + \frac{\mu_0 s D}{\gamma_a} e^{-\gamma_a (x-d)} \tag{S17b}$$

Next, imposing the continuity of tangential fields at $x = d$ directly on their Laplace transformed version, i.e.,

$$\widetilde{h}_z^{(a)}(d^+,s) = \widetilde{h}_z^{(d)}(d^-,s), \qquad \widetilde{e}_y^{(a)}(d^+,s) = \widetilde{e}_y^{(d)}(d^-,s), \tag{S18}$$

gives a set of two equations for the coefficients $C(s)$ and $D(s)$,

$$\begin{bmatrix} C(s) \\ D(s) \end{bmatrix} = \frac{1}{W(s)} \begin{bmatrix} \overline{C}(s) \\ \overline{D}(s) \end{bmatrix}, \tag{S19}$$

where

$$\begin{bmatrix} \overline{C}(s) \\ \overline{D}(s) \end{bmatrix} = \begin{bmatrix} \mu_0 s \frac{1}{\gamma_a} & 1 \\ -\mu_0 s \frac{\cosh(\gamma_{d_2} d)}{\gamma_{d_2}} & \sinh(\gamma_{d_2} d) \end{bmatrix} \begin{bmatrix} H(s)\sin(k_c d) \\ E_1(s)\cos(k_c d) + E_2(s) \end{bmatrix}, \tag{S20}$$

and

$$W(s) = \mu_0 s \left[\frac{\sinh(\gamma_{d_2} d)}{\gamma_a} + \frac{\cosh(\gamma_{d_2} d)}{\gamma_{d_2}}\right] \tag{S21}$$

and

$$H(s) = \frac{s + j\omega_0}{s^2 + \omega_0^2} - \frac{s + j\omega_0 \frac{\epsilon_{d_{r1}}}{\epsilon_{d_{r2}}}}{s^2 + \omega_0^2 \frac{\epsilon_{d_{r1}}}{\epsilon_{d_{r2}}}}, \tag{S22a}$$

$$E_1(s) = \frac{-\mu_0}{\gamma_{d_2}^2} \left[s k_c \frac{s + j\omega_0 \frac{\epsilon_{d_{r1}}}{\epsilon_{d_{r2}}}}{s^2 + \omega_0^2 \frac{\epsilon_{d_{r1}}}{\epsilon_{d_{r2}}}} + \frac{1}{k_c} \left(\beta^2 - j\omega_0 \frac{\epsilon_{d_{r1}}}{\epsilon_{d_{r2}}} \frac{s}{v_{d_2}^2} \right) \right], \tag{S22b}$$

$$E_2(s) = \frac{\mu_0}{\gamma_a^2} \left[\frac{1}{k_c} \left(\beta^2 - j\omega_0 \frac{s}{v_a^2} \right) \cos(k_c d) - sh \sin(k_c d) \frac{s + j\omega_0}{s^2 + \omega_0^2} \right]. \tag{S22c}$$

Solving for $C(s)$ and $D(s)$, inserting into (S14) and (S17a) applying the inverse Laplace transform in Eq.(S7) gives the space time fields $H_z(x, z, t)$ and $E_y(x, z, t)$ (and similarly for $H_x(x, z, t)$ though not used here). The inverse Laplace transform integration in (S7) can be evaluated directly on the region of convergence (ROC) by brute force numerical integration, or by closing the integral with an infinite semi-circle encircling the left (Re$s < 0$) half-space. By doing so, utilizing Jordan's Lemma, the original integration can be replaced by integration around all the singular points in the complex s space. See Figure 1(c) in the main text for illustration of the original and deformed paths.

1.4 Singular points

The inverse Laplace transform involves the evaluation of pole singularities contribution and branch cut singularities. The poles correspond to guided modes, their location provides the modal frequency, while their residue provides the modal amplitude. These are analyzed below. As opposed to the pole contributions, the branch point singularities correspond to the radiation. Their analysis is given in detail in the main text. Returning to the poles, there are several pole singularities that located along the imaginary s axis:

1. Poles at $\gamma_{d_2} = 0$ and $\gamma_a = 0$ i.e., at $s = \pm j\beta v_{d_2}$ and $s = \pm j\beta v_a$.
2. Poles at $s = \pm j\omega_0$ and $s = \pm j\omega_0 \sqrt{\frac{\epsilon_{d_{r1}}}{\epsilon_{d_{r2}}}}$
3. Poles at the zeros of the Woronskian:

$$W(s) = 0. \tag{S23}$$

Following (S21), the Wronskian has two types of zeros, a single pole at $s = 0$ and complex conjugate pairs of poles along the imaginary axis when

$$\sinh(\gamma_{d_2} d)/\gamma_a + \cosh(\gamma_{d_2} d)/\gamma_{d_2} = 0 \tag{S24}$$

denoting them by $s_p^{(i)}$ where $i > 0$ and $i < 0$ indicating poles on the positive and negative sides of the imaginary s-axis, respectively, and with $s_p^{(i)} = j|s_p^{(i)}| = -s_p^{(-i)}$.

Analysis of the contribution of each type of poles can be directly applied. It follows that the poles of items (a)–(b) are removable, with zero residue and thus they have zero contribution to the fields. For the poles of the Wronskian on item (c), it follows that $\text{Res}[C(s); s = 0], \text{Res}[D(s); s = 0] = 0$, hence with no contribution to the fields. Consequently, the only poles with contribution to the fields are those denoted by $s_p^{(\pm i)}$, $i = 1, \ldots i_M$ where i_M depends on d, ϵ_{a_r} and $\epsilon_{d_{r_2}}$. Applying the residue theorem for the evaluation of the inverse Lapalce transform of, for example, H_z in Eq.(S7a) with (S14) gives,

$$H_z(x,z,t)\Bigg|_{\text{poles}} = e^{-j\beta z} \begin{cases} \sum_i \left[\frac{\overline{C}(s)}{W'(s)} \sinh(\gamma_{d_2} x)\right]\Bigg|_{s=s_p^{(i)}} e^{s_p^{(i)} t} & \text{for } 0 \leq x \leq d, \\ \sum_i \left[\frac{\overline{D}(s)}{W'(s)} e^{-\gamma_a (x-d)}\right] + \Bigg|_{s=s_p^{(i)}} e^{s_p^{(i)} t} & \text{for } d \leq x, \end{cases} \tag{S25}$$

where

$$W'(s) = \frac{W(s)}{s} + \mu s^2 \frac{\cosh(\gamma_{d_2} d)}{\gamma_{d_2}^3 v_{d_2}^2} \left[d\frac{\gamma_{d_2}^2}{\gamma_a} - 1 - \gamma_a d + \frac{\gamma_{d_2}^2}{\gamma_a^2} \frac{\epsilon_{a_r}}{\epsilon_{d_{r_2}}} \right], \tag{S26}$$

and $\text{Res}[C(s); s_p] = \frac{\overline{C}(s_p)}{W'(s_p)}$ and $\text{Res}[D(s); s_p] = \frac{\overline{D}(s_p)}{W'(s_p)}$ are the residues at the poles. It is interesting to note that for a given pole index, say i_m, the two associate complex conjugate pole contributions with $\pm i_m$ in (S25) with $s_p^{(i_m)} = \omega_{i_m}$, for $0 \leq x \leq d$ gives the modal contribution:

$$\left\{ \left[\frac{\overline{C}(j\omega_{i_m})}{W'(j\omega_{i_m})} \right] e^{-j(\beta z - \omega_{i_m} t)} + \left[\frac{\overline{C}(-j\omega_{i_m})}{W'(-j\omega_{i_m})} \right] e^{-j(\beta z + \omega_{i_m} |t)} \right\} \sinh(\gamma_{d_2} x), \tag{S27}$$

denoting a forward and backward wave contributions with $\overline{C}/W'$ as the amplitude of the waves and ω_{i_m} is the radian frequency of the modal function. By applying some algebraic manipulations, it can be shown that

$$\left[\frac{\overline{C}(s)}{W'(s)} \right] \Bigg|_{s_p^{(\pm i_m)} = \pm j\omega_{i_m}} = \frac{1}{2} \left[1 \pm \frac{\omega_{i_m}}{\omega_0} \right] \mathcal{C}(\omega_{i_m}^2), \tag{S28}$$

where $\mathcal{C}$ is the wave amplitude which is a symmetric function in s or ω. This factorization suggests that there is a possibility to identify the term in the square brackets as a type of scattering parameter. To that end, define the forward and backward scattering parameters at the modal frequency ω_m by

$$T_m = \frac{1}{2} \left[1 + \frac{\omega_{i_m}}{\omega_0} \right], \qquad R_m = \frac{1}{2} \left[1 - \frac{\omega_{i_m}}{\omega_0} \right] \tag{S29}$$

where T_m and R_m are the forward and backward scattering parameters. Similar algebraic factorization can be applied for $x > d$ which gives the same scattering parameters, T_m and R_m, with $\overline{D}$ instead of

$\overline{C}$ and hence with different wave amplitudes, as expected. Specifically,

$$\frac{\overline{D}(s_p)}{W'(s_p)} = -\left[\frac{1-j\bar{s}_p}{2}\right] \frac{2(\gamma_a d)^3(\gamma_{d_2} d)^3}{\cosh(\gamma_a d)(\epsilon_{a_r}-\epsilon_{d_{r_2}})(k_0 d)^2\bar{s}_p^2\left[\epsilon_{a_r}\epsilon_{d_{r_2}}(k_0 d)^4\bar{s}_p^4+(\beta d)^2(\epsilon_{d_{r_2}}(k_0 d)^2\bar{s}_p^2-\gamma_a d)\right]}$$

$$\times\left\{-\frac{(\epsilon_{a_r}\epsilon_{d_{r_1}}-\epsilon_{a_r}\epsilon_{d_{r_2}})\bar{s}_p^2}{(\epsilon_{a_r}+\epsilon_{a_r}\bar{s}_p^2)(\epsilon_{d_{r_1}}+\epsilon_{d_{r_2}}\bar{s}_p^2)(\gamma_{d_2} d)}\cosh(\gamma_{d_2} d)\sin(k_c d)\right.$$

$$+\frac{\sinh(\gamma_{d_2} d)}{k_c d}\left[-\frac{(\beta d)^2\epsilon_{d_{r_1}}+\epsilon_{d_{r_1}}\epsilon_{d_{r_2}}(k_0 d)^2\bar{s}_p^2}{(\epsilon_{d_{r_1}}+\epsilon_{d_{r_2}}\bar{s}_p^2)(\gamma_{d_2} d)^2}\cos(k_c d)\right.$$

$$\left.\left.+\frac{\epsilon_{a_r}^2(k_0 d)^2\bar{s}_p^2\cos(k_c d)+\left(\epsilon_{a_r}^2(k_0 d)^2\cos(k_c d)+\epsilon_{a_r}(hd)(k_c d)\sin(k_c d)\right)}{(\epsilon_{a_r}+\epsilon_{a_r}\bar{s}_p^2)(\gamma_a d)^2}\right\}\right|_{\bar{s}_p=\bar{s}_p^{(\pm i_m)}=\pm j\frac{\omega_{i_m}}{\omega_0}}$$

$$=\frac{1}{2}\left[1\pm\frac{\omega_{i_m}}{\omega_0}\right]\mathcal{D}(\omega_{i_m}^2), \tag{S30}$$

from which $\mathcal{D}(\omega_{i_m}^2)$, R_m and T_m can easily be identified. Similar extended expression can be obtained for $\mathcal{C}(\omega_{i_m}^2)$.

Additional singularities on the complex s-plane are branch points at $s=\pm jv_a\beta, \pm jv_{d_2}\beta$ and the associate branch cuts. They correspond to radiation which is the main novelty of this work. Their analysis is given in detail in the main text.

2 Dispersive and Lossy Plasmonic slab

The results in the main text are provided for this case. We consider the switching of the parameters of a dispersive and lossy plasmonic slab. We start by formulating the necessary expressions in the time domain. The relative permittivity of a plasmonic slab at frequency ω is given by

$$\varepsilon_r(\omega)=\varepsilon_\infty-\frac{\omega_p^2}{\omega^2-j\omega\Gamma}, \tag{S31}$$

where ϵ_∞ is the limit of $\varepsilon_r(\omega)$ at $\omega\to\infty$, ω_p is the plasma frequency and Γ is a damping rate factor. Recall that

$$\widehat{\mathbf{D}}(\boldsymbol{r},\omega)=\varepsilon_0\widehat{\mathbf{E}}(\boldsymbol{r},\omega)+\widehat{\mathbf{P}}(\boldsymbol{r},\omega), \tag{S32}$$

where $\widehat{\mathbf{D}}$, $\widehat{\mathbf{E}}$ and $\widehat{\mathbf{P}}$ are the electric flux, electric field and polarization vectors, respectively. Inserting (S31) and inverse Fourier transforming from the frequency to the time domain, we obtain the dynamics equation satisfied by the polarization vector,

$$\frac{\partial^2}{\partial t^2}\boldsymbol{P}(\boldsymbol{r},t)+\Gamma\frac{\partial}{\partial t}\boldsymbol{P}(\boldsymbol{r},t)=\varepsilon_0\chi_\infty\frac{\partial^2}{\partial t^2}\boldsymbol{E}(\boldsymbol{r},t)+\varepsilon_0\Gamma\chi_\infty\frac{\partial}{\partial t}\boldsymbol{E}(\boldsymbol{r},t)+\epsilon_0\omega_p^2\boldsymbol{E}(\boldsymbol{r},t), \tag{S33}$$

with $\chi_\infty = \varepsilon_\infty - 1$ where.

Using Eq. (S33) we may introduce time variation in the polarization process. Specifically, assuming that the material model undergoes an abrupt temporal switching of its parameters (e.g., ω_p) at time $t = 0$, it is convenient to use the unilateral Laplace transform representation as the main mathematical analysis tool. Applying the Laplace transform, as defined in (S8) with (S9), to (S33) gives the following expression relating the polarization and the electric field vectors with their initial states at $t = 0^+$,

$$\begin{aligned}\left[\hat{P}(\boldsymbol{r}, s) - \varepsilon_0\chi_\infty\hat{E}(\boldsymbol{r}, s)\right]s^2 + s\Gamma\left[\hat{P}(\boldsymbol{r}, s) - \varepsilon_0\chi_\infty\hat{E}(\boldsymbol{r}, s)\right] - \varepsilon_0\omega_p^2\hat{E}(\boldsymbol{r}, s)\\ = (s+\Gamma)\left[P(\boldsymbol{r}, 0^+) - \varepsilon_0\chi_\infty E(\boldsymbol{r}, 0^+)\right] + \left[P'(\boldsymbol{r}, 0^+) - \varepsilon_0\chi_\infty E'(\boldsymbol{r}, 0^+)\right] \quad \text{(S34)}\end{aligned}$$

where $'$ superscript indicates derivation in time. Furthermore, the polarization and the field on the right hand side are evaluated ant time $t = 0^+$ and therefore use as transient sources for $t > 0$.

In the case discussed here the electric field is polarized in the y direction and the magnetic field in the the z and x directions with

$$\boldsymbol{H}(x, z, t) = \hat{\boldsymbol{z}}h_z(x, t)e^{-j\beta z} + \hat{\boldsymbol{x}}h_x(x, t)e^{-j\beta z} \quad \text{(S35a)}$$

$$\boldsymbol{E}(x, z, t) = \hat{\boldsymbol{y}}e_y(x, t)e^{-j\beta z}. \quad \text{(S35b)}$$

$$\boldsymbol{P}(x, z, t) = \hat{\boldsymbol{y}}p_y(x, t)e^{-j\beta z}. \quad \text{(S35c)}$$

Inserting to Maxwell's and applying the Laplace transform we find that

$$j\beta\tilde{e}_y(x, s) = -\mu_0\left[s\tilde{h}_x(x, s) - h_x(x, 0^+)\right], \quad \text{(S36a)}$$

$$\partial_x\tilde{e}_y(x, s) = -\mu_0\left[s\tilde{h}_z(x, s) - h_z(x, 0^+)\right], \quad \text{(S36b)}$$

$$-\partial_x\tilde{h}_z(x, s) - j\beta\tilde{h}_x(x, s) = \epsilon_0\left[s\tilde{e}_y(x, s) - e_y(x, 0^+)\right] + \left[s\tilde{p}_y(x, s) - p_y(x, 0^+)\right]. \quad \text{(S36c)}$$

Since this is a TE polarization it is convenient to formulate the fields using by using h_z, therefore the corresponding Helmholtz equation is given by

$$\begin{aligned}\partial_x^2\tilde{h}_z(x, s) - \left[\frac{s^2}{v_0^2}(1+\chi(s)) + \beta^2\right]\tilde{h}_z(x, s)\\ = -\Bigg\{\frac{1}{s}\left[\frac{s^2}{v_0^2}(1+\chi(s)) + \beta^2\right]h_z(x, 0^+) + \frac{j\beta}{s}\partial_x h_x(x, 0^+)\\ + \frac{1}{s+\Gamma}\partial_x\left[p_y'(x, 0^+) - \varepsilon_0\chi_\infty e_y'(x, 0^+)\right] - \varepsilon_0(1+\chi_\infty)\partial_x e_y(x, 0^+)\Bigg\}, \quad \text{(S37)}\end{aligned}$$

with $\chi(s) = \chi_\infty + \frac{w_p^2}{s(s+\Gamma)}$, $\partial_x h_x(x, 0^+) = j\beta h_z(x, 0^+)$ and $v_0 = 1/\sqrt{\varepsilon_0\mu_0} = c$ is the speed of light in vacuum. Once solved for $\widetilde{h}_z$, the other transversal field $\widetilde{e}_y$ and h_x can be recovered from (S36) as follows

$$\begin{bmatrix} \widetilde{e}_y(x,s) \\ \\ \widetilde{h}_x(x,s) \end{bmatrix} = \frac{1}{s^2\mu_0\epsilon_0(1+\chi(s))+\beta^2} \begin{bmatrix} \mu s & -j\beta \\ \\ -j\beta & s\varepsilon_0(1+\chi(s)) \end{bmatrix} \times \tag{S38}$$

$$\times \begin{bmatrix} -\partial_x\widetilde{h}_z(x,s) + \varepsilon_0(1+\chi_\infty)e_y(x,0^+) - \frac{1}{s+\Gamma}\left[(1+\chi_\infty)P_y'(x,0^+) + \chi_\infty\left(\partial h_z(x,0^+) + j\beta h_z(x,0^+)\right)\right)\right] \\ \\ \mu_0 h_x(x,0^+) \end{bmatrix}$$

Equation S37 is for a generally plasmonic material. This equation can be used also for the vacuum medium upon, first, noting that there is no polarization component. Furthermore $\chi_\infty = \varepsilon_\infty - 1$ with $\varepsilon_\infty = 1$, $\omega_p, \Gamma \to 0$ giving $\chi(s) = 0$ and the corresponding Helmholtz equation for the vacuum,

$$\partial_x^2\widetilde{h}_z(x,s) - \left[\frac{s^2}{v_0^2} + \beta^2\right]\widetilde{h}_z(x,s) = -\frac{1}{v_0^2}\left[sh_z(x,0^+) - \frac{1}{\mu_0}\partial_x e_y(x,0^+)\right]. \tag{S39}$$

Similarly the expressions for the transversal fields can be given by

$$\begin{bmatrix} \widetilde{e}_y(x,s) \\ \\ \widetilde{h}_x(x,s) \end{bmatrix} = \frac{1}{s^2\mu_0\epsilon_0+\beta^2} \begin{bmatrix} \mu s & -j\beta \\ \\ -j\beta & s\varepsilon_0 \end{bmatrix} \times \begin{bmatrix} -\partial_x\widetilde{h}_z(x,s) + \varepsilon_0 e_y(x,0^+) \\ \\ \mu_0 h_x(x,0^+) \end{bmatrix} \tag{S40}$$

In the following analysis the only properties that are possibly being switched are ω_p and Γ, see e.g. (S31). To that end, assuming that the switching of the medium properties is applied at $t = 0$, the continuity of the field and polarization across the temporal boundary is given by

$$h_{x,z}(x,0^-) = h_{x,z}(x,0^+), \qquad e_y(x,0^-) = e_y(x,0^+) \tag{S41a}$$

$$p_y(x,0^-) = p_y(x,0^+), \qquad p_y'(x,0^-) = p_y'(x,0^+) \tag{S41b}$$

Note that in the case where ε_∞ and μ are switched, then instead of the continuity of the fields in (S41), there will be the continuity of the electromagnetic fluxes.

Next, we insert the expressions of the field components prior to switching at $t = 0^-$. To that end we note that for $t < 0$ the system is basically operating in the time harmonic regime with frequency ω_0, the fields are given by $f(x,t) = \hat{\boldsymbol{f}}(x)e^{j\omega_0 t}$ with $f(x,t) = \hat{\boldsymbol{h}}_z(x,t), \hat{\boldsymbol{h}}_z(x,t), \hat{\boldsymbol{e}}_y(x,t)$. For the slab

region, $0 \leq x \leq d$:

$$\hat{\boldsymbol{h}}_z(x) = \sin(k_c x), \qquad \hat{\boldsymbol{h}}_x(x) = -\frac{j\beta}{k_c}\cos(k_c x), \qquad \hat{\boldsymbol{e}}_y(x) = \frac{j\omega_0\mu_0}{k_c}\cos(k_c x), \tag{S42}$$

and $\hat{\boldsymbol{p}}_y(x) = \varepsilon_0\chi_0(\omega_0)\hat{\boldsymbol{e}}_y(x)$, where $\chi_0(\omega_0) = \chi(s)|_{s=j\omega_0} = (\varepsilon_\infty - 1) - \frac{w_p^2}{\omega_0^2 - j\omega_0\Gamma}$ with ε_∞, ω_p and Γ values for $t < 0$. For the vacuum region, $d \leq x \leq \infty$:

$$\hat{\boldsymbol{h}}_z(x) = e^{-h(x-d)}\sin(k_c d), \quad \hat{\boldsymbol{h}}_x(x) = -e^{-h(x-d)}\frac{j\beta}{k_c}\cos(k_c d), \quad \hat{\boldsymbol{e}}_y(x) = e^{-h(x-d)}\frac{j\omega_0\mu_0}{h}\sin(k_c d). \tag{S43}$$

Addtionaly we have the three dispersion relations

$$k_0^2 = \beta^2 - h^2, \qquad k_0^2(1 + \chi_0(\omega_0)) = \beta^2 + k_c^2, \quad (k_c d)\tan(k_c d) = hd. \tag{S44}$$

Next, inserting the fields at $t < 0$, given in (S42)–(S43), into (S37)–(S40) with the continuity relations in (S41) for $t > 0$ gives the following Helmholtz equation of the $x \geq d$ and $x \leq d$. For $d \leq x \leq \infty$, the Helmholtz equation is given from (S39) by

$$\partial_x^2\widetilde{h}_z(x,s) - \gamma_a^2\widetilde{h}_z(x,s) = -\frac{1}{v_0^2}(s + j\omega_0)e^{-h(x-d)}\sin(k_c d), \qquad \gamma_a = \sqrt{\frac{s^2}{v_0^2} + \beta^2}, \tag{S45}$$

while for $0 \leq x \leq d$ the corresponding Helmholtz equation is given by

$$\partial_x^2\widetilde{h}_z(x,s) - \gamma_d^2\widetilde{h}_z(x,s) = -Q_d\sin(k_c x), \qquad \gamma_d = \sqrt{\frac{s^2}{v_0^2}(1 + \chi(s)) + \beta^2}, \tag{S46}$$

$$Q_d = \left\{\frac{s}{v_0^2}(1 + \chi(s)) + \frac{j\omega_0}{v_0^2}(1 + \chi_\infty) + \frac{1}{s + \Gamma}\left(\frac{\omega_0^2}{v_0^2}(\chi_0(\omega_0) - \chi_\infty)\right)\right\}$$

Solving the two Helmholtz equations for $\widetilde{h}_z(x,s)$ fields in the two regions gives for $d \leq x \leq \infty$

$$\widetilde{h}_z(x,s) = D(s)e^{-\gamma_a(x-d)} + \frac{s + j\omega_0}{s^2 + \omega_0^2}e^{-h(x-d)}\sin(k_c d), \tag{S47}$$

while for $0 \leq x \leq d$ it gives

$$\widetilde{h}_z(x,s) = C(s)\sinh(\gamma_d x) + \frac{Q_d}{\frac{s^2}{v_0^2}(1 + \chi(s)) + k_0^2\varepsilon_{r_1}}\sin(k_c x), \tag{S48}$$

where $\varepsilon_{r_1} = 1 + \chi_0(\omega_0)$ is the relative permittivity for $t < 0$. Note that in both (S47) and (S48) the first term on the right hand side corresponds to the homogeneous solution of the equation while the

second term corresponds to the particular solution of the equations. The unknown coefficients $C(s)$ and $D(s)$ are found by imposing the the spatial continuity relations of the transversal electric and magnetic fields, $\widetilde{e}_y$ and $\widetilde{h}_z$ at the interface $x = d$.

Next imposing the continuity relations of the transversal fields the transversal at $\widetilde{e}_y$ and $\widetilde{h}_z$ at the interface $x = d$ givers the following expression for the coefficients

$$\begin{bmatrix} C(s)) \\ \\ D(s) \end{bmatrix} = \frac{1}{W(s)} \begin{bmatrix} 1 & -\frac{\mu_0 s}{\gamma_a} \\ \\ \sinh(\gamma_d d) & \frac{\mu_0 s}{\gamma_d}\cosh(\gamma_d d) \end{bmatrix} \times \begin{bmatrix} E_d(s)\cos(k_c d) - E_a(s) \\ \\ H_d(s)\sin(k_c d) - H_a(s) \end{bmatrix} \tag{S49}$$

with

$$W(s) = \mu_0 s \left[\frac{\sinh(\gamma_d d)}{\gamma_a} + \frac{\cosh(\gamma_d d)}{\gamma_d}\right], \tag{S50}$$

where

$$H_a(s) = \frac{s + j\omega_0}{s^2 + \omega_0^2}\sin(k_c d) \tag{S51a}$$

$$H_d(s) = \frac{Q_d}{\frac{s^2}{v_0^2}(1 + \chi(s)) + k_0^2\varepsilon_{r1}} \tag{S51b}$$

$$E_a(s) = \frac{\mu_0}{\gamma_a^2}\left[\frac{1}{v_0^2}\frac{j\omega_0 s}{h} - \frac{\beta^2}{h} + sh\frac{s + j\omega_0}{s^2 + \omega_0^2}\right]\sin(k_c d) \tag{S51c}$$

$$E_d = -\frac{\mu_0 s}{\gamma_d^2}k_c\frac{Q_d}{\frac{s^2}{v_0^2}(1 + \chi(s)) + k_0^2\varepsilon_{r1}} \tag{S51d}$$

$$+ \mu_0\frac{j\omega_0\mu_0\varepsilon_0 s}{k_c\gamma_d^2}(1 + \chi_\infty)\left[1 - \frac{j\omega_0\chi_0(\omega_0)}{s + \Gamma}\right] - \frac{\mu_0 s}{k_c\gamma_d^2}\frac{\chi_\infty}{s + \Gamma}\left[k_c^2 + \beta^2\right] - \frac{\beta^2\mu_0}{k_c\gamma_d^2}$$

Once the Helmholtz equations are solved and the coefficients $C(s)$ and $D(s)$ are obtained from (S49), the three field components $\widetilde{h}_{x,z}(x, s)$ and $\widetilde{e}_y(x, s)$ for $0 \leq x \leq d$ and $d \leq x \leq \infty$ following the switching at $t = 0$ can be obtained, they can be inserted into the inverse Laplace transform, (S8), to give the space-time fields $H_{x,z}(x, z, t)$ and $E_y(x, z, t)$ for $t > 0$.

2.1 Singular points

The pole singularities that corresponds to the excitation of guieded mode following the temporal switching can be found by solving $W(s) = 0$. The corresponding residues of the simple poles are the excitation amplitudes of the guided modes, see discussion in Sec. 1.4. Besides the pole singularity the re are also branch point singularities, that corresponds to $\gamma_a = 0$ and $\gamma_d = 0$. This type of singularity is responsible for the transient radiation effect. Note however that the function $\widetilde{h}_z(x, s)$ is continuous

when crossing the branch-cut that corresponds to $\gamma_d = 0$ in the s-plane, and therefore this branch-cut do not contribute to the radiative field. This is a consequence of the fact that $\widetilde{h}_z(x,s)$ is an even function in γ_d, to see that the reader may refer, e.g., to Eq. (S48). The second term on the right hand side in Eq. (S48) is independent of, and therefore even in γ_d. In the first term it is enough to show that $C(s)$ is odd in γ_d, to that end we note that $W(s)$ is odd in γ_d, $E_d(s)\cos(k_c d) - E_a(s)$ is even in γ_d and $-\frac{\mu_0 s}{\gamma_a}\left(H_d(s)\sin(k_c d) - H_a(s)\right)$ is independent of γ_d and thus even.

References

[1] R. E. Collin, *Field Theory of Guided Waves.* Wiley-IEEE Press, 1991.

[2] A. Oppenheim, A. Willsky, and I. Young, *Signals and systems.* Prentice-Hall signal processing series, Prentice-Hall, 1983.
